# Predicting Antibody Neutralization Efficacy in Hypermutated Epitopes Using Monte Carlo Simulations

**DOI:** 10.3390/polym12102392

**Published:** 2020-10-17

**Authors:** Pep Amengual-Rigo, Jorge Carrillo, Julià Blanco, Victor Guallar

**Affiliations:** 1 Barcelona Supercomputing Center (BSC), 08034 Barcelona, Spain; jose.amengual@bsc.es; 2 IrsiCaixa AIDS Research Institute, 08916 Badalona, Spain; jcarrillo@irsicaixa.es (J.C.); jblanco@irsicaixa.es (J.B.); 3 Institut Germans Trias i Pujol, 08916 Badalona, Spain; 4 School of Medicine, University of Vic–Central University of Catalonia, 08500 Vic, Spain; 5 Catalan Institution for Research and Advanced Studies, 08010 Barcelona, Spain

**Keywords:** HIV-1, computational modelling, Monte Carlo simulations, antibody binding efficacy, CD4bs antibodies

## Abstract

Human Immunodeficiency Virus 1 (HIV-1) evades adaptive immunity by means of its extremely high mutation rate, which allows the HIV envelope glycoprotein to continuously escape from the action of antibodies. However, some broadly neutralizing antibodies (bNAbs) targeting specific viral regions show the ability to block the infectivity of a large number of viral variants. The discovery of these antibodies opens new avenues in anti-HIV therapy; however, they are still suboptimal tools as their amplitude of action ranges between 50% and 90% of viral variants. In this context, being able to discriminate between sensitive and resistant strains to an antibody would be of great interest for the design of optimal clinical antibody treatments and to engineer potent bNAbs for clinical use. Here, we describe a hierarchical procedure to predict the antibody neutralization efficacy of multiple viral isolates to three well-known anti-CD4bs bNAbs: VRC01, NIH45-46 and 3BNC117. Our method consists of simulating the three-dimensional binding process between the gp120 and the antibody by using Protein Energy Landscape Exploration (PELE), a Monte Carlo stochastic approach. Our results clearly indicate that the binding profiles of sensitive and resistant strains to a bNAb behave differently, showing the latter’s weaker binding profiles, that can be exploited for predicting antibody neutralization efficacy in hypermutated HIV-1 strains.

## 1. Introduction

Human Immunodeficiency Virus (HIV) causes acquired immunodeficiency syndrome (AIDS), a progressive condition that leads to the failure of the immune system. In 2019, the WHO estimated that 38 million of people were living with AIDS, and nearly 690,000 died of AIDS-related illnesses [1]. The high genetic variability of the virus, induced by a fast replication cycle and a high mutation rate [2,3,4], together with the presence of a latent reservoir of the virus [5,6,7], has impeded the development of vaccines or an effective cure over the past decades [8]. Most treatments against HIV-1 involve the use of combination antiretroviral therapy (cART) for arresting viral replication [9,10]. cART has revolutionized HIV care, improving quality and life expectancy of patients [11,12]. However, life-long cART administration does not cure AIDS, and viral rebound occurs within weeks after interrupting the treatment [13,14,15].

The discovery of broadly neutralizing antibodies (bNAbs) has motivated their use as therapeutic tools against AIDS [16,17,18]. Those antibodies are able to neutralize multiple viral isolates (in some cases, up to 90% of evaluated strains) [19,20,21]. Their high efficacy is achieved by targeting conserved regions of the virus, while also tolerating a set of mutations on the binding interface. Over the past few years, large screening efforts have been made aiming to isolate, describe and characterize potent bNAbs from HIV-1 infected individuals [20,22,23,24,25]. Safety and clinical benefit of using passive transfer of bNAbs have been/are being evaluated in clinical trials [26,27,28,29,30,31,32,33]. Clinical trials reveal that the administration of bNAbs is safe and that current clinical benefit relies on bNAb potency and neutralization breadth. Moreover, it has been also demonstrated that the administration of several complementary bNAbs may be a better therapeutic approach than using individual bNAbs [34].

The major target of bNAbs is the envelope glycoprotein (Env), a large protein complex placed on the surface of virions and infected cells. Env protein consists of a heterotrimer formed by two subunits, gp120 and gp41. Six different regions of the Env protein have been extensively described to elicit potent bNAbs [16]: the CD4 binding site (CD4bs) [35,36,37,38], the V1/V2 apex [39,40,41], the V3 high-mannose loop [42,43,44], the membrane proximal external region (MPER) of gp41 [45,46,47], the gp120–gp41 interface [48,49,50] and the highly glycosylated “silent” face of gp120 [51]. Upon antibody binding, the virus is no longer infectious and is marked for its elimination by the immune system. However, the virus has developed several strategies for escaping antibody recognition. A major resistance mechanism is promoted by the high mutation rate of the virus, which includes a variety of mutations such as insertions, deletions and amino acid substitutions. These diverse mutation events are responsible for reshaping the amino acid composition and, importantly, the glycosylation pattern of the Env protein. Indeed, glycosylation sites have been extensively described as an additional resistance mechanism of the virus [52,53]. However, some regions of the Env protein do not tolerate these changes, since they play a major role during the infectious process. An example is represented by the CD4bs of the gp120, whose major role is to interact with the human CD4 receptor. This protein–protein binding event is critical for the virus and initiates the viral entry into the host cells [54]. Because of the importance of this process, not all mutations on the CD4bs are allowed, since they may compromise the binding to the CD4 receptor. Hence, antibody resistance is reached through a complex equilibrium between acquiring mutations that complicate the antibody recognition, and without compromising the biological role of the altered region.

Being able to predict the efficacy of an antibody towards hypermutated HIV-1 strains is of great interest for the design of therapies against AIDS. Currently, there is no standard computational pipeline for this purpose. However, some computational efforts have been made for highlighting resistance sequence patterns [55,56,57,58,59]. A general strategy is based on taking advantage of previous knowledge, such as experimental binding determinations, to generate a predictive model. Therefore, such statistical models provide a quick overview of putative resistance sequence patterns or mutations that may be even used for diagnostic purposes. However, these methods show strong limitations in the case of poorly characterized antibodies since the availability of experimental data is scarce.

In this work, we designed a computational strategy for predicting the binding efficacy of bNAbs towards multiple HIV-1 epitopes, which could be potentially exploited into other applications. Our strategy consists of modelling the three-dimensional binding process between the antibody and the epitope, by using Protein Energy Landscape Exploration (PELE), a Monte Carlo based software coupled to protein structure prediction [60]. Previous work demonstrated that molecular models of PELE can accurately predict antiretroviral drug efficacy in hypermutated protease HIV-1 strains [61]. By using Monte Carlo simulations, the binding process from small molecules to large protein–protein complexes can be simulated in a relatively short amount of time and computational cost. In this work, we characterized three different anti-gp120 bNAbs (VRC01, NIH45-46 and 3BNC117) against multiple sensitive and resistant gp120 from a diversity of clades. Specifically, we conducted two different studies: (i) evaluation of binding profiles of VRC01 towards a large panel of gp120 strains (45 sensitive and 19 resistant ones), and (ii) evaluation of binding profiles of VRC01, NIH45-46 and 3NBC117 towards nine gp120 strains showing different affinities to those bNAbs. The former study demonstrated that the binding profiles of sensitive and resistant strains towards VRC01 behave differently, which can be exploited for the prediction of antibody efficacy (area under the curve (AUC): 0.84). The latter study demonstrated that the analysis of binding profiles can be used to predict antibody efficacy of different bNAbs towards an HIV-1 strain, which could be exploited to decide the optimum combination of bNAbs for an HIV-1 infected patient. Overall, our results indicate that simulations of the entire binding process of an antibody towards its epitope is an excellent approach to predict the binding efficacy against hypermutated HIV-1 strains.

## 2. Materials and Methods

### 2.1. Data Collection

bNAbs show high neutralization breadth and potency against HIV-1; VRC01 has been described to neutralize ~90% of the experimentally determined HIV-1 strains. Sensitive and resistant strains to those bNabs were determined by ELISA assays, measuring the binding between captured antibodies and monomeric gp120 dissociated from Env pseudovirus proteins. To the best of our knowledge, all 19 experimentally determined resistant strains (half maximal inhibition concentration (IC50) >50 μg/mL) to VRC01 in the literature with an available sequence in GenBank were collected. Those resistant strains represent a diverse set of clades: AC, AE, AG, B, C, CD, D and G. HIV-1 strains coming from similar clades share higher sequence identity than others, and therefore, some bNAbs work better against some particular groups of clades. Therefore, clade identity may play an important role during the prediction. In order to reduce the risk of observing biased results to some sort of clade-peculiarities between both groups, we selected a large set of sensitive strains belonging to similar clades to the resistant ones. Hence, we selected 45 strains sensitive to VRC01 representing the following clades: A, AC, AD, ACD, AE, AG, B, BC, C, CD, D. Table S1 shows an overview of the features of all selected strains, accounting for strain name, clade identity, IC50 (μg/mL) and residues that are placed in the interface region upon VRC01 binding: the loop D, the CD4 loop, the β20-β21 region and the β23-V5-β24-α5 region. As can be observed, the residues placed in the interface region are unique for each strain.

Moreover, we also aimed to evaluate if the binding affinity of different anti-gp120 bNAbs can be predicted towards the same set of gp120 strains, which could be exploited for selecting the optimal combination of bNAbs for personalized care. In order to do this, we evaluated 9 gp120 HIV-1 strains showing different binding affinities [35,36] to the three anti-gp120 bNAbs evaluated in this work: VRC01, NIH45-46 and 3BNC117. Table S2 represents the experimental determinations of those strains towards the three bNAbs.

### 2.2. Modeling of Three-Dimensional gp120 Structures

From a structural point of view, only a minority set of the gp120 HIV-1 strains evaluated in this work have been characterized three-dimensionally. This limitation was overcome using Prime from Schrödinger to generate homology models of all gp120 strains [62,63]. As a template structure, we used the 93TH057 gp120 strain co-crystallized on the Protein Data Bank (PDB) accession: 3NGB [64]. This entry contains the bound conformation of the 93TH057 gp120 to the VRC01 antibody, with a resolution of 2.68 Å.

Once the three-dimensional models of the gp120 were constructed, all possible N-glycosylation sites were also modelled. Since the most common N-glycosylation observed in all available three-dimensional gp120 structures consists of a covalent modification of asparagine with an N-acetyl-glucosamine (NAG) residue, we assume that all surface exposed asparagine satisfying the putative linear glycosylation motif N-X-T/S were NAG-glycosylated.

### 2.3. Simulating the Binding Process with PELE

**Figure 1:**
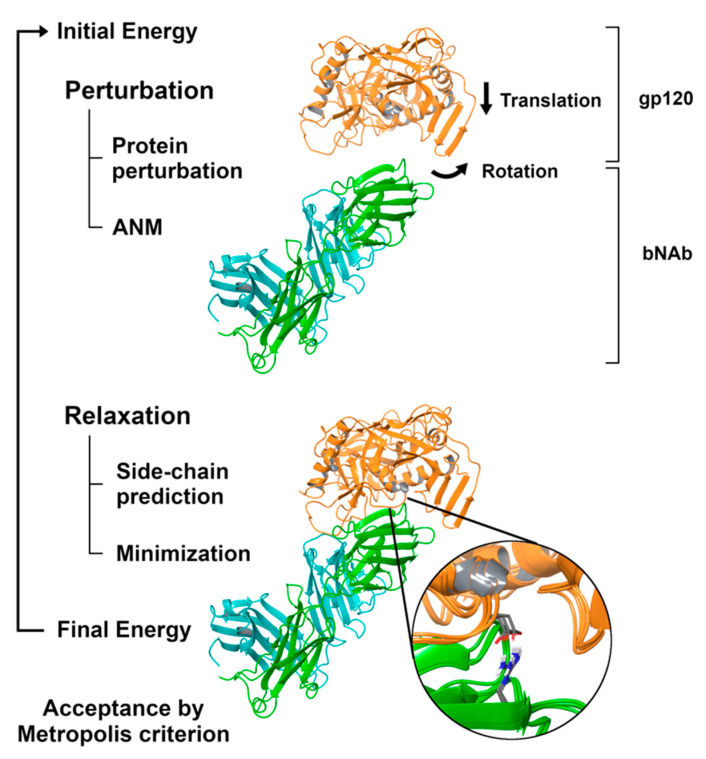
Graphical representation of a Protein Energy Landscape Exploration (PELE) step for protein–protein simulations. PELE consists of two phases: perturbation and relaxation. During the former, a protein is perturbed (random translations and rotations), and a sampling protocol based anisotropic network model (ANM) is performed to account for backbone motions. During the latter, a side-chain protocol and a minimization step are performed to generate energetically stable conformations. The entire movement is accepted according to a Metropolis criterion.

To the end of simulating the binding process between bNAbs and gp120, we used the PELE software. PELE is a Monte Carlo method, typically linked to protein-ligand studies, that combines a stochastic approach with protein structure prediction techniques. Simulations use the unbound conformations between the gp120 and the bNAbs as starting points. All gp120 strains were placed in the same position and 10 Å away in the vertical axis from the observed bound conformation in the template structures. This shift allows the necessary motions of the gp120 to simulate the binding process. Figure 1 represents a graphical scheme of a PELE simulation step.

Each simulation step consists of applying random translation and rotations to a group (in this case, the gp120), perturbations of the protein backbone (using normal modes), side-chain prediction and minimization. More in detail, PELE simulations start by applying very small translations and rotations to the gp120. After these perturbations, PELE initiates a protein structure sampling protocol based on anisotropic network models (ANM) that allows protein backbone motions. Then, a side-chain sampling protocol of the residues involved in the protein–protein interaction is conducted to predict energetically stable side-chain conformations. Finally, a global minimization process is performed. The final movement is accepted or rejected under a Metropolis criterion, which is based on the total energy of the system using the OPLS2005 force field with a variable dielectric generalized surface Born implicit solvent [65]. If the simulation step is accepted, the new conformation will be the starting pose of the following step. However, if the simulation step is rejected, the starting pose of the following step will be the last accepted along the simulation. By performing many PELE steps over time, the binding process of the gp120 to a bNAb can be simulated. Indeed, the nature of the Metropolis criterion will favor the acceptance of energetically favorable conformations. Hence, our hypothesis was that the dynamics of reaching deeply bound conformations would be slower (or impossible) for resistant strains, while it would be facilitated for the sensitive ones. Therefore, the population analysis of such PELE simulations would indicate the likelihood of the binding efficacy of an antibody towards its epitope. In order to obtain enough sampling to perform this study (this is, obtaining thousands of intermediate conformations between the initial undocked pose to the final conformation), each PELE simulation was carried out using 144 independent trajectories (each running on a computing core) during 48 h.

### 2.4. Population Analysis of PELE Simulations

**Figure 2:**
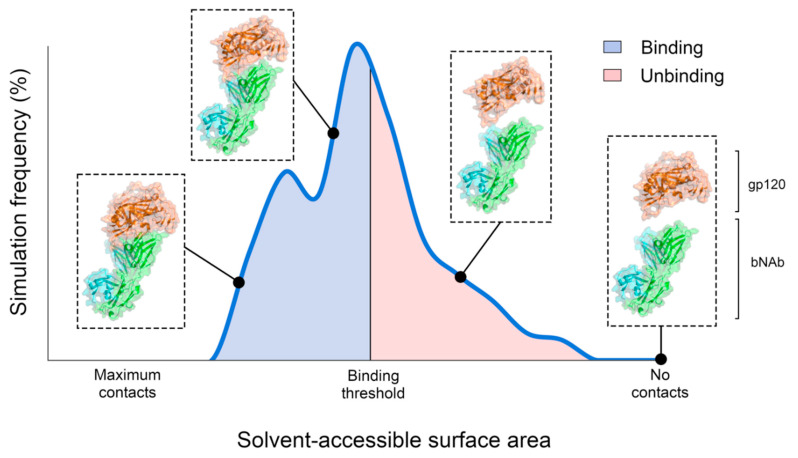
Graphical representation of a PELE simulation based on the frequency of protein–protein contacts. PELE simulations start from unbinding conformations, and over time, protein–protein contacts between the epitope and the antibody can be formed. Solvent-accessible surface area (SASA) of the gp120 indicates the amount of contacts formed with the antibody. Relative binding affinities can be determined by applying a SASA threshold along the simulation by using Equation (1).

Population analysis of the PELE simulations was conducted to evaluate the binding process of the gp120 strains towards the antibodies. These analyses were mainly based on the solvent-accessible surface area (SASA). SASA is a measure that indicates the proportion of the gp120 that is accessible to the solvent. The gp120 of the initial conformation has a SASA value of 1, since both proteins are placed 10 Å away in the vertical axis from the bound conformation). Along the PELE simulation, the gp120 is randomly perturbed, and only the energetically favorable conformations are accepted. Therefore, the gp120 may move closer to the antibody and, in turn, result in a decrease in the SASA value. Hence, a complex three-dimensional binding process of an epitope towards an antibody can be simplified into the distribution of the SASA values along the entire PELE simulation (Figure 2).

In this way, simulations enriched with structures lacking protein–protein contacts (higher SASA values) indicate that such epitopes may be resistant (or less efficient) towards the selected antibody, while the other way indicates the opposite. In fact, predicted relative binding affinity of an epitope can be quantitatively determined by defining an SASA threshold representing two simulation states: binding and unbinding. Hence, the predicted relative binding affinity can be estimated through the log likelihood of the amount of PELE steps belonging to each simulation state (Equation (1)). Hence, gp120 strains predicted to be sensitive towards an antibody are those with negative relative binding affinity scores, while the other way indicates the opposite.

(1) $$\mathrm{Predicted}\;\mathrm{relative}\;\mathrm{binding}\;\mathrm{affinity}=\;-\log\left(\frac{\mathrm{Amount}\;\mathrm{of}\;\mathrm{binding}\;\mathrm{events}}{\mathrm{Amount}\;\mathrm{of}\;\mathrm{unbinding}\;\mathrm{events}}\right)$$

## 3. Results

### 3.1. Evaluating Binding Profiles of Sensitive and Resistant Strains to VRC01

**Figure 3:**
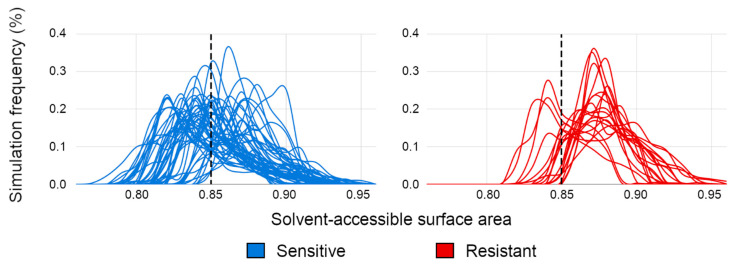
Frequency of solvent-accessible surface area (SASA) determination of PELE simulations in a set of 45 sensitive (blue) and 19 resistant (red) Human Immunodeficiency Virus 1 (HIV-1) gp120 strains. The dashed line indicates a SASA value threshold of 0.85, used for defining a binding and unbinding cut-off event.

A set of 45 sensitive and 19 resistant HIV-1 gp120 strains from a diverse set of clades were selected to construct a predictive model of the VRC01 antibody binding efficacy (Table S1). Figure 3 contains the population distribution of the SASA measurements of those strains in the PELE simulations. As can be observed, sensitive strains (blue) are predicted to achieve higher amounts of contacts (reflected by reaching lower SASA values) than the resistant strains (red). These results indicate that simulating the binding process may provide insights on antibody binding efficacy.

**Figure 4:**
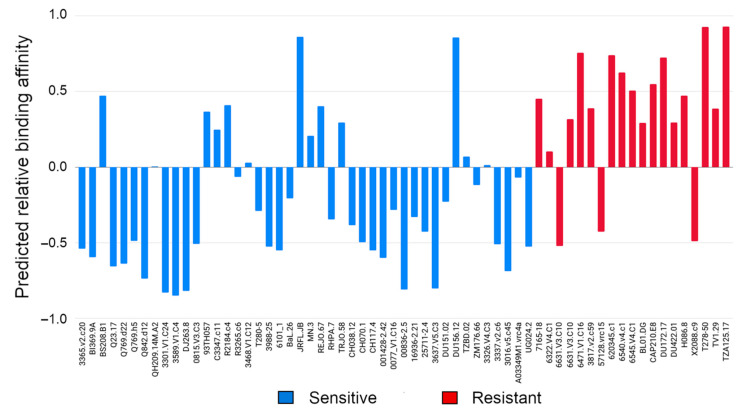
Predicted relative binding affinities of 45 sensitive (blue) and 19 resistant (red) HIV-1 gp120 strains towards VRC01, extracted from PELE simulations (Figure 3). Most of the sensitive strains are correctly predicted (33 out of 45), while most of the resistant strains are also predicted as such (16 out of 19).

Aiming to quantitatively determine the predicted relative binding affinity of those gp120 strains, we defined two states: binding and unbinding. The threshold that defines both states was selected by computing the mean of the SASA values from the maximum frequency peaks in all sensitive strains, resulting in a mean SASA value of 0.85 (Figure 3). Hence, the predicted relative binding affinities of all selected gp120 strains towards VRC01 were computed as described in Equation (1) and are depicted in Figure 4. Most of the sensitive strains (blue) show negative values and are therefore correctly predicted to be sensitive (33 out of 45), while most of the resistant strains (red) are predicted to be as such by the positive predicted affinity (16 out of 19).

**Figure 5:**
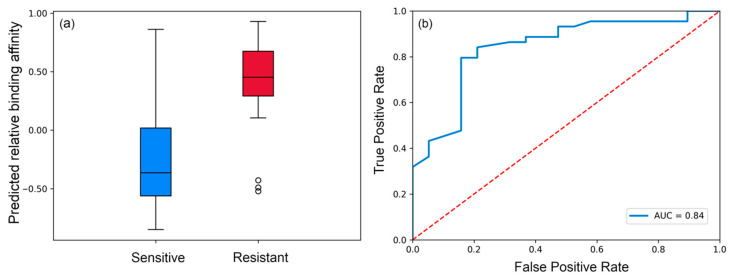
Statistics of predicted affinities of 45 sensitive and 19 resistant HIV-1 gp120 strains towards VRC01. (**a**) Distribution of the predicted binding affinities; (**b**) Receiver operator characteristic (ROC) curve achieving an area under the curve (AUC) of 0.84.

Since the experimental binding determinations are heterogeneous, in the sense that there exists a strong binding (0.001 μg/mL < IC50 < 5 μg/mL) or it does not exist (IC50 > 50 μg/mL), we aimed to focus our analysis based on the binary classification, rather than performing correlations between experimental and predicted binding. This decision is supported by two main limitations of the experimental data: (i) most of the sensitive strains show a potent binding (IC50 < 0.2 μg/mL) and a few of them show midterm potency (maximum IC50 value for sensitive strains < 5 μg/mL) (Figure S1), (ii) the determinations of the resistant strains are undetermined (>50 μg/mL) (Figure S2). Both limitations challenge the success of a correlation assay, and therefore the analysis was based on differentiating sensitive and resistant patterns from the binary classification point of view. The distribution and the receiver operator characteristic (ROC) curve of the predicted relative binding affinities to VRC01 is illustrated in Figure 5. As can be observed, the predicted affinity distribution (left panel) of both sensitive and resistant groups differs substantially. Moreover, the ROC curve analysis indicated a strong predictive power for determining antibody binding efficacy, achieving an AUC of 0.84.

### 3.2. Evaluating Binding Affinity of VRC01, NIH45-46 and 3BNC117 to Multiple Viral Isolates

A set of nine different gp120 strains representing a variety of HIV-1 clades were selected to evaluate the binding efficacy of three different bNAbs recognizing the CD4bs in gp120: VRC01, NIH45-46 and 3BNC117. As reported in Table S2, those bNAbs show different binding affinities to the selected strains. Therefore, we aimed to evaluate if the binding profiles of sensitive and resistant strains observed in VRC01 could be extrapolated to other bNAbs, as a general predictive tool for predicting antibody binding efficacy.

Despite the fact that the three antibodies share high sequence similarity (especially VRC01 and NIH45-46), their amino acid composition differs, especially in the regions that are located on the interface with the gp120. Indeed, the variable regions of NIH45-46 and 3BNC117 contain several insertions and deletions in comparison with VRC01. An example of that is the CDR3 loop of the heavy chain, which is one of the most important gp120 binding regions of the antibody. Compared to VRC01, NIH45-46 contains an insertion of four residues in that region, while 3BNC117 contains a deletion of a length of two residues. Therefore, a fixed SASA threshold for all bNAbs for considering the binding and the unbinding state cannot be used, since the amount of contacts that those antibodies can make is different. As mentioned in previous sections, the template structure for the generation of the homology models was PDB: 3NGB. In this accession, the binding mode of VRC01 was characterized with the 93TH057 gp120 strain. The other bNAbs were also characterized with the same strain, under the following accession PDB codes: 3U7Y (NIH45-46) [66] and 4JPV (3BNC117) [67]. Hence, the SASA values of the bound conformations can be determined, and the optimum threshold for defining the bound and the unbound state can be estimated for NIH45-46 and 3BNC117 (Equation (2)). In this way, we defined an optimum SASA threshold of 0.83 for NIH45-46, and 0.86 for 3BNC117.

(2) $${\mathrm{bNAb}}_{\mathrm{PELE}}=\;\mathrm{VRC}01_{\mathrm{PELE}}-\left(\mathrm{VRC}01_{\mathrm{BOUND}}-{\mathrm{bNAb}}_{\mathrm{BOUND}}\right)\;$$

Equation (2) gives the optimum SASA threshold for defining binding and unbinding events for uncharacterized bNAbs (bNAb_PELE_). VRC01_PELE_ stands for the SASA value of 0.85 computed for VRC01 in previous sections. VRC01_BOUND_ stands for the SASA calculation of a reference gp120 for the bound (crystal structure) complex with VRC01. bNAb_BOUND_ stands for the SASA calculation of a reference gp120 for the bound (crystal structure) complex with the uncharacterized bNAb.

**Figure 6:**
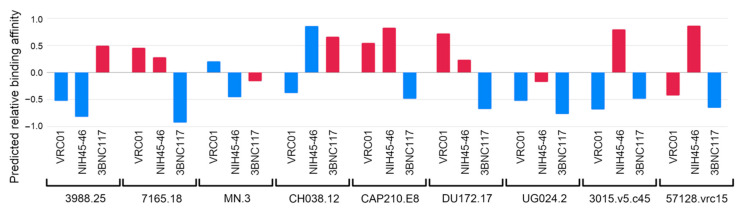
Predicted relative binding affinities of 9 different HIV-1 gp120 strains towards VRC01, NIH45-46 and 3BNC117 using PELE simulations. Most of the sensitive strains (blue) are correctly predicted (12 out of 14, negative predicted affinity), while most of the resistant strains (red) are also predicted as such (10 out of 13, positive predicted affinity).

PELE simulations of the NIH45-46 and 3BNC117 bNAbs were performed under the same conditions as the simulations with VRC01. Predicted relative binding affinities were computed by using the optimum thresholds of each bNAb (Figure 6). As can be observed, the binding affinity of each gp120 strain is predicted for the three bNAbs. A predicted affinity lower than zero indicates a sensitive prediction, while the other way indicates the opposite. Each color represents the current experimental binding affinity of the gp120 strain for that specific antibody (Table S2). In this way, sensitive strains are colored in blue and resistant strains in red. Hence, blue colored predictions with a score lower than zero are correctly classified as sensitive, while red colored predictions with a score higher or equal to zero are correctly classified as resistant. Our results indicate that most of the experimental determinations to the three bNAbs are correctly predicted for sensitive (12 out of 14) and resistant (10 out of 13) HIV-1 strains.

### 3.3. Structural Basis of Resistance Mechanisms

**Figure 7:**
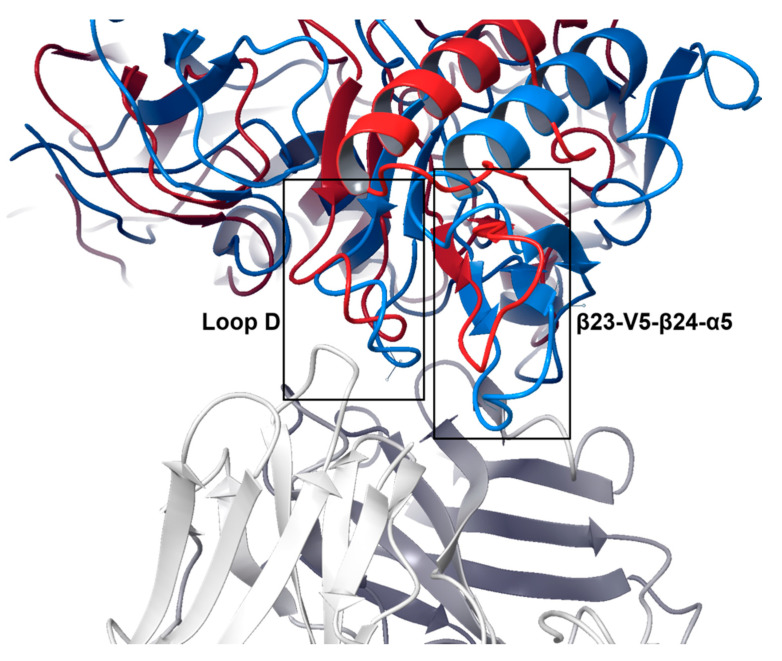
Representative binding mode of sensitive (Q482.d12, blue) and resistant (620345.c1, red) gp120 strains to VRC01. Depicted gp120 strains illustrate the representative structure of the maximum SASA frequency peaks of the PELE simulations (as shown in Figure 3). Analysis of the PELE predictions indicate that the antibody is unable to accommodate the loop D and the β23-V5-β24-α5 region of the resistant gp120 strains.

After determining that the binding profiles of resistant gp120 strains are weaker than the sensitive ones, we aimed to investigate the three-dimensional basis of such behavior. For each antibody and gp120 strain, we analyzed the conformations generated by PELE from the maximum SASA frequency peaks (as observed in Figure 3 for VRC01). We observed that most gp120 glycoproteins derived from the resistant strains adopt a similar unbound conformation that, compared to the sensitive ones, is unable to strongly bind the antibody (Figure 7). This effect is mostly driven by the inability of the antibody to accommodate two important contact regions of gp120: the loop D and the β23-V5-β24-α5 region. In fact, some point mutations in those regions have been associated with antibody resistance in previous studies [35,36,68], such as position 279 (N/D-279-K/E/Q/R), 280 (N280D), 281 (A281T), 456 (R456W), 458 (G458D). Several resistant strains evaluated in this work contain some of those mutations in those positions, such as TV1.29, DU422.01, TZA125.17, 6471.V1.C16, 620345.c1, BL01.DG, H086.8. All gp120 strains containing mutations known to drive antibody resistance to VRC01 were correctly predicted as such (Figure 4), corroborating the experimental findings of previous studies [36,68]. Resistant gp120 variants that do not contain any of the above-mentioned mutations display other mutations in loop D and in the β23-V5-β24-α5 region, that may potentially contribute to resistance to antibody binding. Importantly, both loop D and β23-V5-β24-α5 regions are close in space (Figure 7), and mutations in one of these regions could affect the conformational dynamics of the other, altering accommodation with the antibody.

## 4. Discussion

After almost four decades of worldwide research against AIDS, there is no available cure or vaccine. Recent discoveries of potent bNAbs that are able to neutralize up to 90–98% of the tested HIV-1 strains have opened the door to the use of alternative immunotherapies based on the passive transfer of antibodies [18]. Individual bNAbs or their combination have been/are being evaluated in multiple clinical trials, and potent bNAb variants are continuously being described [69]. Therefore, predicting the binding efficacy of an antibody towards individual hypermutated HIV-1 strains is of great interest for personalized immunotherapies. Currently, there is not any standard pipeline for this task, and most efforts have focused on the analysis of the available sequences, which may highlight some resistant patterns (for instance, a specific amino acid substitution in the protein–protein interface with the binding antibody). These analyses require large amounts of data to generate a predictive model, which limits their applicability to very-well characterized bNAbs and to similar previously described resistant mutations in a similar way as large datasets are applied to genotypically identify resistance to current antiretrovirals (https://hivdb.stanford.edu/). However, HIV Env protein shows higher variability and plasticity than any other viral target [70], adding complexity to this potential approach. Moreover, sequence-based analyses usually use the primary sequence of the epitope without including the structural information of the protein–protein complex. The structural data contains a higher amount of information than linear sequences, which may be crucial for unveiling binding affinity patterns of bNAbs, such as for instance, the position and orientation of the amino acids in the three-dimensional space or the presence of non-covalent interactions (such as hydrogen bonds, salt bridges, hydrophobic interactions, etc.). Indeed, simulation of the three-dimensional binding process may also provide more information, since it includes the needed conformational changes required for the correct coupling of the antibody to the epitope. Such simulations, however, come at a significant increase in computational cost.

In this work, we took advantage of well-established and computationally efficient PELE technology to simulate the binding process of multiple bNAbs to several epitopes that are known to be sensitive and resistant to them. PELE’s Monte Carlo simulations can be especially interesting for evaluating the binding process of such large complexes, since they are relatively computationally cheap and fast. Our results demonstrate that the binding process of an antibody towards its epitope can be successfully simulated by generating thousands of intermediate complexes from an unbound to bound conformation. The population analysis of the binding profiles revealed that sensitive and resistant strains behave differently. More in detail, sensitive strains achieved higher amounts of contacts with the bNAb than resistant ones. From those binding profiles, we defined a contact threshold to define two states, binding and unbinding, and we computed a predicted affinity score based on the amount of intermediate structures generated by PELE in the two states. Hence, simulations containing more intermediate structures in the binding state are predicted as sensitive ones, while the other way indicates the opposite. We simulated the binding process of VRC01 to 45 sensitive and 19 resistant gp120 strains from a diverse set of clades, and we observed that the resistant strains show weaker binding patterns than the sensitive ones. Certainly, our pipeline correctly classified 33 out of 45 sensitive strains and 16 out of 19 resistant strains, with an AUC of 0.84. Moreover, we aimed to extrapolate these findings to other anti-gp120 bNAbs, NIH45-46 and 3BNC117. In order to perform this extrapolation, we simulated with PELE the binding process of those bNAbs against nine different gp120 HIV-1 strains from different clades showing different binding affinities. Our results indicate that most of the sensitive and resistant strains are predicted correctly as such (12 out of 14 sensitive cases and 10 out of 13 resistant cases). As in most modelling efforts, we simplify a complex process aiming at large scale applicability. Herein, the structures were generated with homology modelling and the internal dynamics of both proteins largely reduced, simplifications that could be the source of the false positives/negatives observed. Concretely, we hypothesized that the modeling of the V5 region could play a major role in this topic, since it is one of the most hypermutated regions of the gp120 containing large amounts of insertions and deletions. Next, we aimed to elucidate the three-dimensional determinants of antibody resistance in those antibodies. After the analysis of the conformations generated by PELE, we concluded that resistance is driven by the inability of the antibody to accommodate mutations in the loop D and in the β23-V5-β24-α5 region, which impairs the binding. These results are in line with previous studies, where some point mutations in those regions have been reported to drive antibody resistance. Overall, our results demonstrate that PELE simulations are an excellent approach for characterizing antibody binding efficacy of hypermutated gp120 to bNAbs. Such detailed information could open the door for future antibody engineering, aiming at bypassing resistant patterns.

## Supplementary Materials

The following are available online at https://www.mdpi.com/2073-4360/12/10/2392/s1, Table S1: Overview of the selected gp120 HIV-1 strains from a diversity of clades, Table S2: Sensitive and resistant strains towards three anti-CD4bs antibodies. Figure S1: Experimental IC50 determinations of VRC01 sensitive strains. Figure S2: Experimental IC50 determination of VRC01 resistant strains.
