# Supplementary material for: Predicting Antibody Neutralization Efficacy in Hypermutated Epitopes Using Monte Carlo Simulations
Source: Polymers (Basel). 2020 Oct 17;12(10):2392. doi: 10.3390/polym12102392 (PMC7602999; doi:10.3390/polym12102392)
Supplement: Supplementary file 1 [file polymers-12-02392-s001.pdf]

Supplementary material

Predicting antibody neutralization efficacy in hypermutated epitopes using Monte Carlo simulations

Pep Amengual-Rigo [1], Jorge Carrillo [2], Julià Blanco [2,3,4] and Victor Guallar [1,5,*]

[1] Barcelona Supercomputing Center (BSC), 08034 Barcelona, Spain; jose.amengual@bsc.es
[2] IrsiCaixa AIDS Research Institute, 08916 Badalona, Spain; jcarrillo@irsicaixa.es (J.C.); jblanco@irsicaixa.es (J.B.)
[3] Institut Germans Trias i Pujol, 08916 Badalona, Spain
[4] School of Medicine, University of Vic–Central University of Catalonia, 08500 Vic, Spain
[5] Catalan Institution for Research and Advanced Studies, 08010 Barcelona, Spain
[*] Correspondence: victor.guallar@bsc.es; Tel.: +34-934137727

Strain name	Clade	IC50 (VRC01)	Loop D	CD4 loop	β20-β21	V5-β24-α5
3365.v2.c20	A	0.025	ENITNNAKN	FNGS-SGGDLEITTHSF	KQIINMWQRAGQAIYA	GLILTRDGGNNNN---SSNETFRPGGGDMRDNW
BI369.9A	A	0.08	ENITNNVKT	FTNS-SGGDLEVTTHSF	RQIINMWQRTGQAMYA	GLLLTRDGGGN--N--NTNETFRPGGGDMRDNW
BS208.B1	A	0.005	ENITNNAKN	FANP-LGGDLEITTHSF	KQIINMWQRVGQAMYA	GIILTRDGGY---N--NTNETFRPGGGDMRDNW
Q23.17	A	0.084	ENITNNAKI	FANS-SGGDLEITTHSF	KQIINMWQRAGQAMYA	GLLLTRDGGKD--N--NVNETFRPGGGDMRDNW
Q769.d22	A	0.012	ENITNNAKN	FNNS-LGGDLEITTHSF	KQIINMWQRVGQAIYA	GLILTRDGGIIN----STEETFRPGGGDMRDNW
Q769.h5	A	0.026	ENITNNAKN	FNNS-LGGDLEITTHSF	KQIINMWQRVGQAIYA	GLILTRDGGIINST--DTDEIFRPGGGDMRDNW
Q842.d12	A	0.013	ENITNNAKT	FANS-TGGDLEITTHSF	KQIINMWQRVGQAMYA	GLILTRDGGNT-N---STRETFRPGGGDMRDNW
QH209.14M.A2	A	0.008	ENFTNNAKN	FTNS-SGGDIEITTHSF	KQIINMWQRVGRAIYA	GLILTRDGGDDE----NNTETFRPGGGNMRDNW
3301.V1.C24	AC	0.155	ENLTDNVKT	FKPS-SGGDPEITTHSF	RQIINMWQGVGRAIYA	GLLLTRDGGSDG-N--STKETFRPGGGDMRDNW
3589.V1.C4	AC	0.132	KNLTDNAKT	FAKH-SGGDLEITTHSF	KQIVNAWQRVEQAIYA	GLLLTRDGGGNNS---SENETFRPGGGDMRDNW
3468.V1.C12	AD	0.063	ENITNNAKT	FANA-SGGDLEVTTHSV	KQIINMWQRTGQAMYA	GLLLTRDGGDTS----SMNETFRPGGGDMRDNW
0815.V3.C3	ACD	0.042	ENITNNGKN	FNKS-AGGDLEVTTHSF	KQIINMWQRVGQAMYA	GLILTRDGGSNT----NASETFRPGGGDMRDNW
93TH057	AE	0.027	ENLTNNAKT	FQPP-SGGDLEITMHHF	KQIINMWQGTGQAMYA	GILLTRDGGANN----TSNETFRPGGGNIKDNW
C3347.c11	AE	0.094	ENLENNAKT	FRPP-LGGDLEITMHHF	KQIINMWQGTGQAMYA	GILLTRDGGNNS----ADNETFRPGGGNIKDNW
R2184.c4	AE	0.068	ENLTDNTKT	FQPP-SGGDLEITMHHF	KQIINMWQRVGQAMYA	GILLTRDGGTNTS---KNNETFRPGGGNIKDNW
R3265.c6	AE	0.054	ENITNNAKT	FRPP-SGGDLEITMHHF	KQIINMWQGVGQAMYA	GILLTRDGGDDG----ATNETFRPVGGNIKDNW
DJ263.8	AG	0.108	EDITNNAKN	IFANSSGGDIEITTHSF	KQIVNMWQKVGLAMYA	GLLLTRDGGSNNS----TNETFRPGGGDMRDNW
3988.25	B	0.552	ENLTDNAKT	FNQS-SGGDAELVMHSF	KQIINMWQEVGKAMYN	GLLLTRDGGNNNNT--NTTETFRPEGGNMKDNW
6101.1	B	0.069	ENLTDNAKT	FNQS-SGGDLEIVMHTF	KQIINRWQEVGKAMYA	GLLLTRDGGDNN----NTIETFRPGGGDMRDNW
BaL.26	B	0.037	ENFTNNAKI	FKHS-SGGDPEIVTHSF	KQIINMWQEVGRAMYA	GLLLTRDGGPE--D--DKTEVFRPGGGDMRDNW
JRFL.JB	B	0.02	DNFTNNAKT	FNHS-SGGDPEIVMHSF	KQIINMWQEVGKAMYA	GLLLTRDGGINEN----GTEIFRPGGGDMRDNW
MN.3	B	0.016	ENFTDNAKT	FNPS-SGGDPEIVMHSF	KQIINMWQKVGKAMYA	GLLLTRDGGEDT-DT-NDTEIFRPGGGDMRDNW
REJO.67	B	0.046	ENFTDNAKI	FNQS-SGGDPEVTMHTF	KQIINMWQRVGKAIYA	GLILTRDGGNSSL---SSPEIFRPGGGDMRDNW
RHPA.7	B	0.045	ENFTNNVKN	FAPS-SGGDPEIVMHSF	RQIINMWQEVGKAMYA	GLLLTRDGGVDT-----TKETFRPGGGNMKDNW
TRJO.58	B	0.069	KNFSDNAKI	FNQP-SGGDPEVTMHSF	KQIINRWQEVGKAMYA	GLLLTRDGGKTA--N-NTTEFFRPGGGNMKDNW
CH038.12	BC	0.386	ENLTDNAKI	FESS-SGGDLEITTHSF	KQIINMWQGVGQAMYA	GLLLTRDGGRSNET--NDTETFRPEGGNMKDNW
CH070.1	BC	1.67	ENLTNNAKT	FAPH-SGGDLEITTHSF	RQIVRMWQQVGQAMYA	GLLLVRDGGNI--N--RTNETFRPEGGDMRDNW
CH117.4	BC	0.021	ENLTDNVKT	FTSS-SGGDLEIATHSF	KQIINMWQEVGQAMYA	GLLLERDGGRDIN----NTEIFRPGGGDMKNNW
001428-2.42	C	0.026	ENLTDNVKT	FTSS-SGGDLEITTHSF	KQIINMWQEVGRAMYA	GLLLVRDGGKN-----NNTEIFRPGGGDMRDNW
0077_V1.C16	C	1.03	ENITDNVKT	FQPPSPGGDLEITTHSF	KQIINMWQGVGRAMYA	GILLTRDGSETNDGNTTETEIFRPGGGDMRDNW
00836-2.5	C	0.242	KKLDDNANT	FNSS-SGGDLEITTHSF	KQVINLWQEVGRAIYA	GLLLVRDGGNHEEA--NTTETFRPGGGNMRDNW
16936-2.21	C	0.046	ENLTDNVKT	FNSS-SGGDLEITTHSF	KQIVNMWQKVGRAMYA	GLLLVRDGGPD-----NVTEIFRPGGGDMRDNW
25711-2.4	C	0.793	ENITDNAKT	FNSS-SGGDLEITTHSF	KQIINMWQEVGRAVYA	GILLTRDGGRGEEVK-NDTETFRPGGGNMKDNW
3637.V5.C3	C	4.6	ENITDNVKT	KQPS-PGGDLEITMHSF	KQIINMWQEVGRAMYA	GLLLVRDGGISNGTD-NKNETFRPGGGDMRNNW
DU151.02	C	2.51	ENLTNNIKT	FKPP-SGGDLEVTTHSF	KQIINMWQKVGRAMYA	GLLLTRDGGK--N---TTNEIFRPGGGNMKDNW
DU156.12	C	0.088	ENLTDNIKT	FEPP-SGGDLEITTHSF	KQIINMWQGVGRAMYA	GLLLTRDGGGNVTEI-NRTEIFRPGGGNMKDNW
TZBD.02	C	0.07	KNLTNNVNT	FKPS-SGGDLEITTHSF	KQIVNMWQEVGRAMYA	GLLLVRDGGGES----NETEIFRPGGGDMRDNW
ZM176.66	C	0.055	ENLTDNAKT	FEPH-SGGDLEITTHSF	KQIVNMWQGVGRAMYA	GLLLTRDGGNDD----NDTETFRPGGGDMRDNW
3326.V4.C3	CD	0.087	ENLTNNVKN	FEPA-LGGDPEITTHTF	KQIINMWQGVGKAMYA	GLLLTRDGNNSH-------ETFRPGGGDMGDNW
3337.v2.c6	CD	0.026	ENITNNAKT	FQPS-SGGDPEITEHTF	KQIINRWQGVGKAMYA	GLLLTRDGGNT------SEEIFRPGGGDMRDNW
3016.v5.c45	D	0.193	ENISNNAYN	FKPS-AGGDAEITTHSF	KQIINMWQGVGKAMYA	GLILTRDGGNT--S--DDHETFRPGGGNMKDNW
A03349M1.vrc4a	D	2.94	ENLTNNAKI	FKPS-SGGDPEITTHSF	KQIINMWQGVGKAMYA	GLLLTRDGGGNE-S--SQNETFRPGGGDMRDNW
UG024.2	D	0.157	ENITNNAKI	FKPS-SGGDPEITTHSF	KQIVNMWQGVGKAMYA	GLLLTRDGGN--T---SQNETFRPGGGDMRDNW
6540.v4.c1	AC	>50	EHIGNSAKN	FKNS-SGGDIEITTHSF	KQIINMWQRAGQAIYA	GLILTSDYGNN-N---SDNEIFRPTGGDMRDNW
6545.V4.C1	AC	>50	EDITNSVKN	FKNS-SGGDIEITTHSF	KQIINMWQRAGQAIYA	GLILTSDYGNR-S---SDNETFRPTGGDMRDNW
620345.c1	AE	>50	EDITKNTKT	FQPP-SGGDLEVTTHHF	KQIVRMWQGVGQSMYA	GILLTSDGDGGP-T--ADNETFRPAGGDMRDNW
242-14	AG	>50	ENISNNGKT	FTNH-SGGDLEVTTHSF	KQIINMWQRVGQAMYA	GLLLTRDGGFRNDTN-ETYEAFRPGGGDMRDNW
T278-50	AG	>50	KNISANAKT	FTKP-SGGDLEITTHSF	KQIINMWQTVGQAMYA	GLLLTRDGEAG--K--STNETFRPIGGNMRDNW
BL01.DG	B	>50	KNFTQNAET	FNPPIRGGDPEIVMHNF	KQIINLWQKVGKAMYA	GLLLTRDGGKNGT---EGTEIFRPIGGNMRDNW
H086.8	B	>50	ENFTKNEKT	FNQS-TGGDPETAMFTF	RQIVNMWQRIGKAMYA	GLLLTRDGDKN-N---KSTEVFRPIGGEMRDNW
7165.18	B	>50	ENFTDNVKT	FMQH-SGGDPEIVTHTF	KQIINMWQGVGKAMYA	GLLLTRDGGENRTD--NGTEIFRPGGGNMRDNW
CAP210.E8	C	>50	ENISNNVKT	FAPP-VGGDLEITTHSF	RQIINMWQEVGRAMYA	GLLLTRDGGENKTEN-NDTEIFRPGGGDMKDNW
DU172.17	C	>50	ENLTNNAKI	FAPS-SGGDLEITTHSF	KQIIRMWQGVGQAMYA	GLLLTRDGGKE--K--NDTETFRPGGGDMRDNW
DU422.01	C	>50	ENLTNNIKT	FEPS-SGGDLEVTTHSF	KQIINMWQEVGRAMYA	GLLLTWDGGEN-----STEGVFRPGGGNMKDNW
TV1.29	C	>50	ENLTENTKT	QFKPHAGGDIEITMHSF	KQIVRMWQGVGQAMYA	GILLTRDGGFNTT---NNTETFRPGGGDMRDNW
TZA125.17	C	>50	ENLTNNAKT	FKPAVVGGDLEITTHSF	KQFVNMWQRVGRAMYA	GLLLTWDGGNNT----NGTETFRPGGGDMRDNW
6322.V4.C1	C	>50	ENLTNNAKI	FQPH-SGGDLEVVTHSF	KQIINMWQEVGRAMYA	GLLLERDGGKDN-N---MTEIFRPGGGDMRDNW
6471.V1.C16	C	>50	KDLNNTGNT	FSPH-PGGDLEVTMHSF	KQIINMWQGVARAMYA	GLLLTWDGDKTSND--PDTDVFRPGGGNMKDNW
6631.V3.C10	C	>50	ENLTNNAKI	FESH-SGGDLEITTHSF	KQIINMWQEVGRAMYA	GILLTRDGGPN-----STNETFRPEGGDMRNNW
3817.v2.c59	CD	>50	ENVTNNAKT	FSPS-SGGDPEITTHSF	KQIVNMWQGVGRAMYA	GLLLTRDGGLNT----SNNETFRPGGGDMRDNW
57128.vrc15	D	>50	ENLTNNAKI	FNAS-SGGDPEITTHSF	KQIINMWQGVGKAMYA	GLLLTRDGGGADNN--RQNETFRPGGGDMRDNW
X2088.c9	G	>50	ENLTNNAKV	FNSP-AGGDLEITTHSF	KQIVRMWQRVGQAMYA	GLLLTRDGVNDTHD--KENETFRPTGGDMRDNW

Table S1: Overview of the selected gp120 HIV-1 strains from a diversity of clades.

Strain name	Clade	VRC01	NIH45-46	3BNC117
3988.25	B	Sensitive	Sensitive	Resistant
7165.18	B	Resistant	Resistant	Sensitive
MN.3	B	Sensitive	Sensitive	Resistant
CH038.12	BC	Sensitive	Sensitive	Resistant
CAP210.E8	C	Resistant	Resistant	Sensitive
DU172.17	C	Resistant	Resistant	Sensitive
UG024.2	D	Sensitive	Resistant	Sensitive
3016.v5.c45	D	Sensitive	Resistant	Sensitive
57128.vrc15	D	Resistant	Resistant	Sensitive

Table S2: Sensitive and resistant strains towards three anti-CD4bs antibodies.

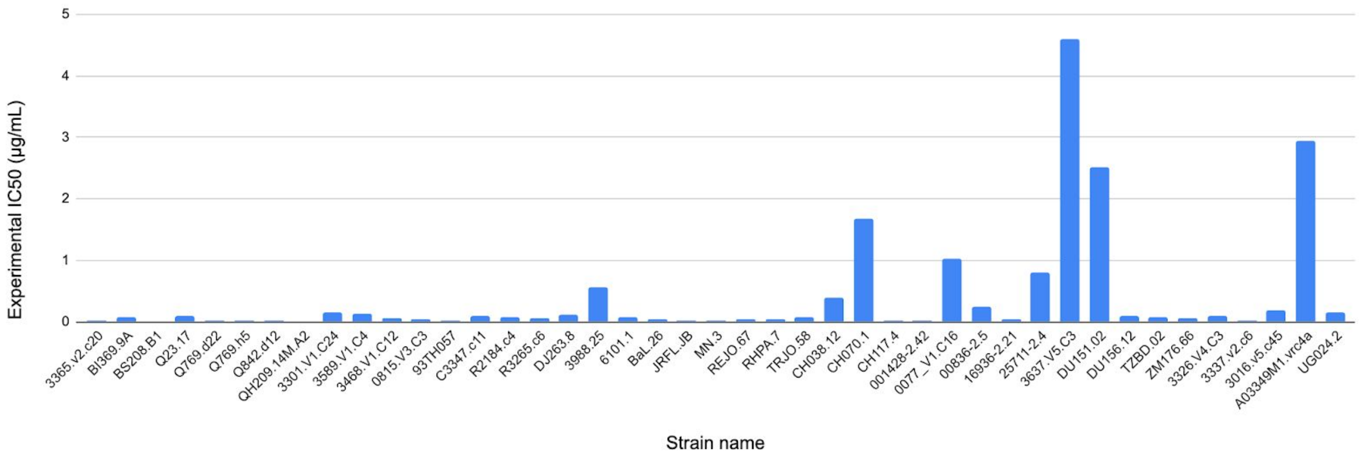

Figure S1. Experimental IC50 determinations (µg/mL) of the VRC01 sensitive strains evaluated in this work. As can be observed, most of all binding determinations have values < 0.2 µg/mL.

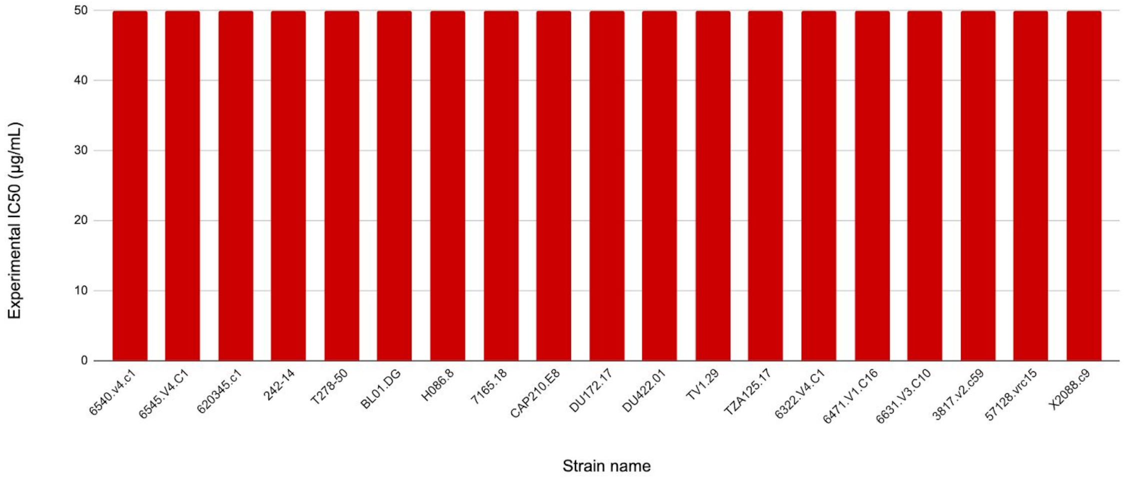

Figure S2. Experimental IC50 determinations (µg/mL) of the VRC01 sensitive strains evaluated in this work. A numerical value 50µg/mL was assumed, since the current experimental data is ">50µg/mL" for all of those strains.
